# Lung Congestion Severity in Kidney Transplant Recipients Is Not Affected by Arteriovenous Fistula Function

**DOI:** 10.3390/jcm11030842

**Published:** 2022-02-05

**Authors:** Krzysztof Letachowicz, Anna Królicka, Andrzej Tukiendorf, Mirosław Banasik, Dorota Kamińska, Tomasz Gołębiowski, Magdalena Kuriata-Kordek, Katarzyna Madziarska, Oktawia Mazanowska, Magdalena Krajewska

**Affiliations:** 1 Department of Nephrology and Transplantation Medicine, Wroclaw Medical University, 50-556 Wrocław, Poland; miroslaw.banasik@umw.edu.pl (M.B.); dorota.kaminska@umw.edu.pl (D.K.); tomasz.golebiowski@umw.edu.pl (T.G.); magdalena.kuriata-kordek@umw.edu.pl (M.K.-K.); katarzyna.madziarska@umw.edu.pl (K.M.); oktawia.mazanowska@umw.edu.pl (O.M.); magdalena.krajewska@umw.edu.pl (M.K.); 2 Faculty of Medicine, Wroclaw Medical University, 50-367 Wrocław, Poland; a.krolicka@student.umw.edu.pl; 3 Department of Social Medicine, Wroclaw Medical University, 50-367 Wroclaw, Poland; andrzej.tukiendorf@umw.edu.pl

**Keywords:** vascular access, arteriovenous fistula, kidney transplantation, lung ultrasound, B-lines

## Abstract

Lung ultrasound is a bedside technique for the assessment of pulmonary congestion. The study aims to assess the severity of lung congestion in kidney transplant recipients (KTR) in relation to arteriovenous fistula (AVF) patency. One hundred fifty-seven patients at least 12 months after kidney transplantation were recruited to participate in a cross-sectional study. Apart from routine visits, lung ultrasound at 28 typical points was performed. The patients were assigned to either AVF+ or AVF− groups. The mean number of lung ultrasound B-lines (USBLs) was 5.14 ± 4.96 with no differences between groups: 5.5 ± 5.0 in AVF+ and 4.8 ± 4.9 in AVF−, *p* = 0.35. The number and proportion of patients with no congestion (0–5 USBLs), mild congestion (6–15 USBLs), and moderate congestion (16–30 USBLs) were as follows: 101 (64.7%), 49 (31.4%), and 6 (3.8%), respectively. In multivariate analysis, only symptoms (OR 5.90; CI 2.43,14.3; *p* = 0.0001), body mass index (BMI) (OR 1.09; CI 1.03,1.17; *p* = 0.0046), and serum cholesterol level (OR 0.994; CI 0.998,1.000; *p* = 0.0452) contributed significantly to the severity of lung congestion. Lung ultrasound is a valuable tool for the evaluation of KTR. Functioning AVF in KTR is not the major factor affecting the severity of pulmonary congestion.

## 1. Introduction

Lung ultrasound (LUS) is an easy-to-learn and reproducible bedside technique for the assessment of pulmonary congestion. Initially, it was used in intensive care units [1,2,3]; later on, it was found that LUS comets (B-lines) represent a practical and simple way for cardiologists to visualize extravascular lung water [4]. The number of B-lines is a predictor and treatment goal in heart failure patients [5,6]. The severity of lung congestion is also a measure of survival in dialysis patients [7]. In a recent multicenter trial, lung ultrasound-guided hemodialysis treatment was compared with regular care. In the active arm, recurrent episodes of decompensated heart failure and cardiovascular events were reduced [8]. Lung ultrasound also has great potential as a screening and monitoring tool in SARS-CoV-2 infection. Intensive care units can use LUS to identify poorly-aerated areas of the lungs and monitor the effects of ventilation changes on lung aeration [9]. Recent years have also seen a growing interest in the use of LUS in nephrology [10]. Apart from dialysis, of which the value is well-documented, LUS has also been used to assess the condition of patients with nephrotic syndrome or acute kidney injury [11,12,13]. Nonetheless, experience with lung ultrasound in renal transplant recipients (KTR) is rather limited. A small study performed in the early postoperative period reported slight changes in the number of B-lines; however, the total number of B-lines remained low [14]. Bearing in mind that functioning arteriovenous fistula (AVF) might have a deleterious systemic effect [15,16,17], we performed a cross-sectional study to assess the severity of lung congestion in the KTR cohort in relation to vascular access function.

## 2. Materials and Methods

**Figure 1:**
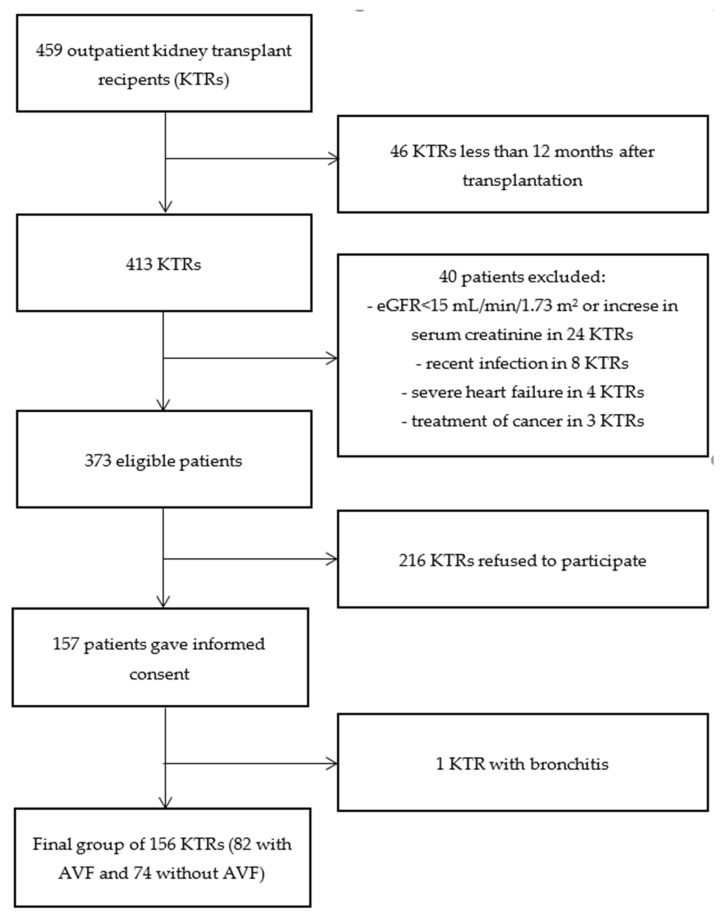
Study flowchart. AVF—arteriovenous fistula; eGFR—estimated glomerular filtration rate.

The study protocol was approved by the Institutional Ethical Committee at Wroclaw Medical University (KB-43/2020) and registered in ClinicalTrial.gov (NCT04478968). Between July and October 2020, 157 patients at least 12 months after kidney transplantation (KTx) were recruited to participate in a cross-sectional study. They were selected from a group of 459 patients who were scheduled for a routine visit in the transplantation outpatient clinic. The inclusion criteria were as follows: age 18 years and over, at least 12 months after kidney transplantation, stable kidney function, and ability to provide informed consent. The following exclusion criteria applied: eGFR (estimated glomerular filtration rate) < 15 mL/min/1.73 m^2^; a severe infection within 3 months preceding the testing, an increase in serum creatinine concentration > 0.5 mg /dL within 3 months before the testing, untreated cancer, and severe heart failure (NYHA IV). The study flowchart is presented in Figure 1. Written informed consent was obtained from all patients. As previously planned, the patients were divided into two groups: with patent AVF, and without vascular access. Clinical data were collected through a direct interview and from medical records. They included information on demographics, comorbidities, vascular access history and function, and routine laboratory data. The presence of heart disease was defined as a history of coronary artery disease, valvular heart disease, or arrhythmia. Patients with dyspnea, peripheral edemas, or crackles on auscultation were classified as symptomatic.

### 2.1. Ultrasound Assessment

All ultrasound examinations were performed by the same nephrologist using the Samsung HS50 system. A linear probe was used for vascular access while a convex probe was used for lung ultrasound tests. Arteriovenous fistula flow (Qa) was assessed as per the guidelines [18]. In the cross-sectional study, we investigated the effect of functioning AVF on the number of ultrasound B-lines (USBLs) in the KTR group. Lung ultrasound was performed in line with the previously described twenty-eight scanning site scheme. After a few minutes of rest in the supine position, ultrasound scanning was performed, proceeding from the second to the fourth intercostal space on the left chest side (to the fifth on right) along the parasternal, mid-clavicular, anterior axillary, and mid-axillary lines. The number of vertical reverberation artifacts (USBLs) was counted at each scanning site and added. The total number of USBLs from all 28 sites gave a score that represents the severity of pulmonary congestion. Lung ultrasound tests were performed by the same experienced nephrologist who has received formal training via a remote web-based program and has been certified by an expert [8,19,20,21]. The number of USBLs was compared in subgroups in relation to AVF patency (AVF−, AVF+, Qa < 1500 mL/min, Qa > 1500 mL/min), presence of symptoms, and history of heart disease. Qa > 1500 mL/min was considered as a high flow promoting the development of heart failure [22].

### 2.2. Study Group

The studied cohort was described previously [23] and its characteristics are summarized in Table 1. It comprised 99 (63%) males and 88 (37%) females; the mean age was 55.3 ± 11.5 years, the mean time from KTx was 108.3 ± 64.5 months, and the mean serum creatinine concentration was 1.46 ± 0.52 mg/dL. Most patients (147 out of 157, 94.2%) were on hemodialysis before transplantation. The majority of them (151 of 157, 96.2%) received a graft from a deceased donor. Due to signs of bronchitis, one patient was not included in the analysis as inflammation is an amplifier of lung congestion [24]. The most common maintenance immunosuppression regimen included a corticosteroid, calcineurin inhibitor, and mycophenolate, and was administered in 119 (76.3%) KTRs. All patients received calcineurin inhibitor; mainly tacrolimus, which was administered in 128 patients (82%). Corticosteroids were administered in 148 (94.9%), mycophenolate in 127 (81.4%), mTOR inhibitor in 7 (4.5%), and azathioprine in 6 patients (3.8%).

### 2.3. Statistical Analysis

Statistical analyses were performed using Statistica 13.3 (StatSoft, Tulsa, OK, USA), the “cluster” R package [25] within the R statistical platform [26]. Categorical variables were presented as proportions and compared with the Chi-Square test. Continuous data were presented as mean and standard deviations. Initially, statistical differences between the groups of patients indexed as AVF+ and AVF− were determined using the appropriate parametric and non-parametric tests. An analogous statistical procedure was performed for the groups of patients divided into the “no congestion”, “mild congestion”, and “moderate congestion” groups. Then, ordinal logistic regression was used to assess the impact of selected risk factors on the ordinal scale of USBL severity, and the observed results of the cause-and-effect relationship were expressed using the classic odds ratios (ORs). Moreover, besides the regressive relationships between the clinical factors, the non-regressive method was also considered in statistical analysis. An original taxonomic method by Marczewski and Steinhaus [27] was used instead of traditional linear modeling, the statistical effectiveness of which has been proven in clinical investigations in several previous studies [28]. In general terms, this statistical method consists of finding relatively heterogeneous clusters of patients and homogeneity within them, i.e., obtaining the highest possible extra-group variance while maintaining the lowest possible intra-group variability. The division of set elements is made with the use of arbitrarily chosen classification variables (risk factors). Then, using specially adapted dendrograms, we matched the clinical responses to defined patient groups and tested their statistical relations with the collected explanatory variables using the appropriate (parametric and non-parametric) tests. *p* < 0.05 was considered significant.

## 3. Results

### 3.1. Lung Ultrasound B-Lines

The mean number of USBLs was 5.14 ± 4.96 with no differences between AVF+ and AVF−, groups: 5.5 ± 5.0 and 4.8 ± 4.9, *p* = 0.35. Lung congestion severity was similar in AVF− patients and AVF+ patients with Qa <1500 mL/min and >1500 mL/min, 4.7 ± 4.8, 5.6 ± 5.0, and 5.1 ± 5.2, *p* = 0.4086, respectively. The number and proportion of patients with no congestion (0–5 USBLs), mild congestion (6–15 USBLs), and moderate congestion (16–30 USBLs) were as follows: 101 (64.7%), 49 (31.4%), and 6 (3.8%), respectively. The characteristics of the groups are presented in Table 1 and Table 2. We did not diagnose any patients with severe pulmonary congestion (>30 USBLs).

**Figure 2:**
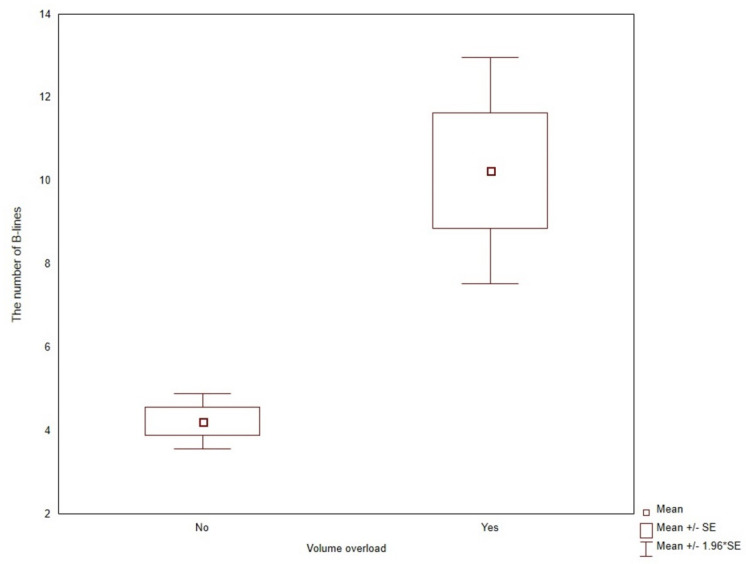
The number of B-lines in patients with and without symptoms of volume overload.

**Figure 3:**
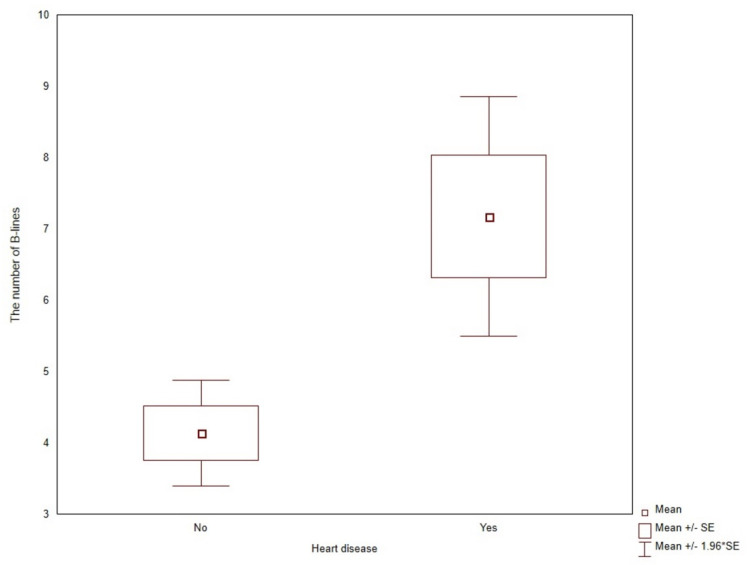
The number of B-lines in patients with and without a diagnosis of heart disease.

The number of USBLs was higher for symptomatic patients (10.25 ± 6.8 versus 4.22 ± 3.92, *p* < 0.0001) and patients with diagnosed heart diseases (7.17 ± 6.18 versus 4.13 ± 3.87, *p* = 0.0081), respectively (Figure 2 and Figure 3).

### 3.2. Predictors of Lung Congestion

Multiple variables related to the number of USBLs were identified with ordinal logistic regression (Table 3). In multivariate analysis, only symptoms (OR 5.90; CI 2.43,14.3; *p* = 0.0001), body mass index (BMI) (OR 1.09; CI 1.03,1.17; *p* = 0.0046) and serum cholesterol level (OR 0.994; CI 0.998,1.000; *p* = 0.0452) contributed significantly to the severity of lung congestion. 

### 3.3. Classification of Patients with Taxonomy

**Figure 4:**
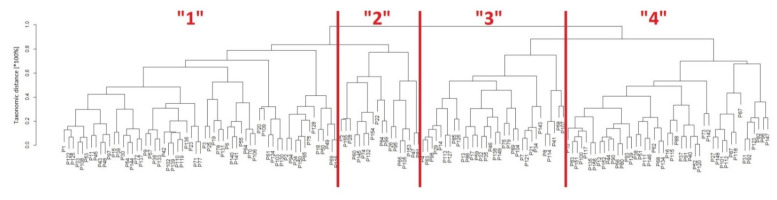
Patient classification tree (dendrogram) based on classification variables (Charlson comorbidity index, BMI, and cholesterol). Four groups of patients were distinguished (Clusters “1”, “2”, “3”, and “4”).

Afterward, several three-dimensional sets of the analyzed classification risk factors were studied before finding the significant data structures with clinical events (we limited the taxonomy to three-variable systems so as not to complicate the interpretation of the patient classification). An “optimal” patient classification for assessing possible correlations was determined for the Charlson comorbidity index, BMI, and cholesterol, giving the best data representation of other clinical factors of the study material collected. Based on these classifiers, a hierarchical classification tree (dendrogram) was created by establishing the distance between the set elements in the three-dimensional data space and projecting it onto a two-dimensional data plane (Figure 4), allowing patient sets to be grouped into their subsets with similar properties according to the clinical features assumed.

In Figure 4, specific patients (coded by “*p*” numbers) are hierarchically aggregated from up to down in separate branches represented by the dendrogram leaves. Following the scree plot of observed heights, the four main families (Clusters “1”, “2”, “3”, and “4”) of the studied patients were distinguished in the classification tree based on their Charlson comorbidity index, BMI, and cholesterol levels. Then, following the “*p*” numbers, patient identification was performed and descriptive statistics of the established clusters were prepared using a one-way ANOVA test (parametric and non-parametric) with adjusted Bonferroni *p*-values correction (Table 4).

### 3.4. Lung Ultrasound B-Lines in Clusters of Patients

**Figure 5:**
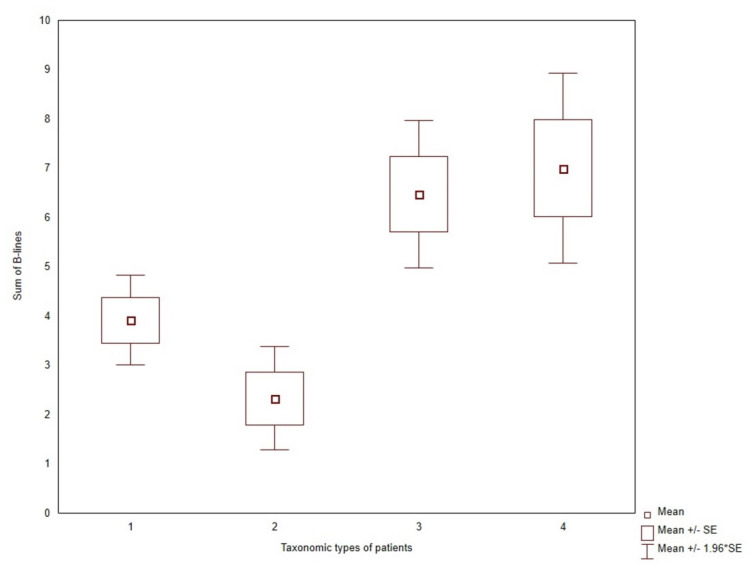
The number of B-lines in the subgroup of patients classified with taxonomy into four clusters.

A significantly higher number of USBLs (Figure 5) was found in patients from clusters 3 and 4 (3.9 ± 3.6, 2.3 ± 2.3, 6.5 ± 4.3, and 7.0 ± 6.6, *p* = 0.0003), respectively. The patients from clusters 3 and 4 were older (51.0 ± 10.1, 45.6 ± 10.2, 56.2 ± 10.9, and 64.4 ± 7.5, *p* < 0.0001) and had higher prevalence of heart disease in comparison to clusters 1 and 2 (18%, 17%, 47%, 51%, *p* = 0.0004). The proportion of patients with functioning AVFs was also higher but was on the threshold of statistical significance. Nonetheless, the total time of AVF functionality was the longest in patients from cluster 4.

## 4. Discussion

The primary finding of this study is that the number of USBLs was low in stable KTRs. No difference in the severity of lung congestion between AVF+ and AVF− patients was found, even after the inclusion of access flow. In hemodialysis patients, the reported number of USBLs was much higher. In a study by Zoccali, only 10% of patients had a USBL number at or below 5; additionally, mild congestion was found in another 31% of patients. More than 15 USBLs were detected in 59% of patients [7]. In patients with heart failure, the mean number of USBLs was 36.6 ± 34.2 and the proportion of patients with at least 15 US-BLs was 68% [29]. The results of our study reflect some important findings regarding the severity of lung congestion. The number of USBLs is related to transplanted kidney function and comorbidity burden—particularly history and symptoms of cardiovascular disease. It emphasizes the importance of heart and renal interactions with the lungs as a target organ. Further studies are needed to confirm the lung water cascade [30] similar to that observed in patients with hypertension and heart failure, both with preserved and reduced ejection fraction. Dwyer et al. found that the proportion of patients with at least 3 USBLs was the lowest in hypertensive patients and the highest in patients with heart failure with reduced ejection [31]. The results of our study cannot be easily compared with those obtained in the study by Dwyer et al. since the latter used a different scanning scheme based on eight zones. However, a comparison of the eight and twenty-eight site scores has shown a high interrelation of both lung ultrasound protocols [32]. Therefore, we can assume that USBL severity in our cohort falls between hypertensive and heart failure with preserved ejection fraction. The next valuable finding is the impact of obesity on the severity of pulmonary congestion. The relation of high BMI and the number of USBLs underlines the role of weight gain after transplantation as a cardiovascular risk factor. However, a high BMI was also observed in cluster 1, a group of patients with low USBLs. Our observation is different from the findings of Palazzuoli et al. They found that in patients admitted to hospital due to heart failure, the number of USBLs was lower in obese patients. An inverse correlation of USBLs and BMI was observed [33,34]. The interrelation between obesity and the severity of lung congestion deserves more attention. We have also found that the severity of lung congestion is greater in KTx patients diagnosed with heart diseases. This is in line with observations in the general population. As mentioned previously, the severity of USBLs increases from hypertension to heart failure, with a preserved ejection fraction and heart failure with reduced ejection fraction [31]. It is also unsurprising that a higher number of B-lines was noticed in patients with dyspnea, edema, or crackles on auscultation; however, in hemodialysis patients, lung crackles and peripheral edema poorly reflected lung congestion detected with ultrasound [35]. Based on personal experiences and results of previous trials, we think that the number of USBLs can be used as a surrogate marker of cardiovascular risk and a predictor of outcomes in KTRs as well. Its diagnostic, therapeutic, and prognostic value has been demonstrated in acute and chronic heart failure [5,36,37,38,39,40] with USBLs > 15 as a cut-off value for a poor outcome. In our cohort, moderate congestion (> 15 USBLs) was found only in six (3.8%) patients. The proportion is much lower than reported for dialysis and stable heart failure patients [7,41,42,43]. We can assume that prognosis for the majority of stable KTx patients is good. Finally, using taxonomic classification, we were able to identify subgroups of patients with more severe lung congestion. The question is whether the future outcomes of patients from various clusters will be different. 

While recent years have seen an increase in the amount of available data on deleterious effects of functioning AVF [44,45], the majority of studies included symptomatic patients with high-flow AVFs. Our study mainly included patients with mild or no symptoms and with distal vascular access, unlike studies assessing the trajectory of eGFR after fistula formation and closure. In observational studies, the rate of change of the eGFR was reduced after AVF creation [46] and accelerated when AVF was ligated in patients after KTx [47]. Moreover, the patients’ perspectives cannot be ignored. Many of them are not convinced about allowing the elective removal of the vascular access [23,48]. The decision to close an AVF after transplantation should always be made on a case-by-case basis, taking into account vascular access, cardiac and kidney graft function, and potential complications [49]. If in doubt, flow restriction or AVF reconstruction may be the optimal solution [50,51]. 

Lung ultrasound is an easy and rapid bedside technique providing ample clinically useful information [52]. It is worth using in daily practice. The major limitation of this study is the lack of echocardiographic assessment. However, it was shown previously that USBLs are positively correlated with E/e’ and negatively correlated with ejection fraction [53]. Other limitations include the observational nature of the study, single-center origin, and a limited number of participants. Furthermore, the study was conducted during the COVID outbreak, which may have affected the patients’ decisions regarding participation in the study.

## 5. Conclusions

The use of lung ultrasound is a valuable tool for the evaluation of kidney transplant recipients. Functioning AVF in KTR is not the major factor affecting the severity of pulmonary congestion. The approach to the KTR should be multifactorial and focused on numerous cardiovascular, metabolic, and immunologic factors.

## Figures and Tables

**Table 1:** Baseline characteristics by AVF function.

Characteristics	AVF Present *n* = 82	AVF Absent *n* = 74	*p*-Value
Sum of B-lines	5.5 ± 5.0	4.8 ± 4.9	0.3507
Age (years)	56.4 ± 10.9	54.1 ± 12.2	0.2137
Male, *n* (%)	56 (68.3%)	42 (56.7 %)	0.1397
BMI (kg/m^2^)	27.0 ± 4.3	26.6 ± 5.4	0.5612
Serum creatinine concentration (mg/dL)	1.45 ± 0.50	1.47 ± 0.55	0.7658
eGFR (mL/min/1.73m^2^)	53.5 ± 14.7	53.2 ± 17.1	0.9802
Duration between study visit and transplantation (months)	90 ± 57	129 ± 66	0.0001
Duration between study visit and RRT initiation (months)	144 ± 81	174 ± 72	0.0154
First transplantation, *n* (%)	68 (82.9%)	64 (86.5%)	0.8753
Diabetes mellitus, *n* (%)	19 (23.2%)	15 (20.3%)	0.6641
Heart disease, *n* (%)	32 (39%)	20 (27%)	0.1141
Charlson comorbidity index	4.5 ± 2.0	4.3 ± 1.6	0.4179
Smoking, current or previous, *n* (%)	38 (46.3%)	34 (45.9%)	0.9114
Medications, *n* (%)			
Steroids, calcineurin inhibitor, mycophenolate, *n* (%)	66 (80.5%)	53 (71.6%)	0.2663
Antihypertensive, *n* (%)	75 (91.5%)	66 (89.2%)	0.8346
Statins, *n* (%)	33 (40.2%)	26 (35.1%)	0.6229
Antiplatelet/anticoagulants, *n* (%)	24 (29.3%)	14 (18.9%)	0.1879

BMI—body mass index; eGFR—estimated glomerular filtration rate; RRT—renal replacement therapy; AVF—arteriovenous fistula.

**Table 2:** Characteristics of patients in relation to the severity of lung congestion.

Characteristics	No Congestion *n* = 101	Mild Congestion *n* = 49	Moderate Congestion *n* = 6	*p*-Value
Sum of B-lines	2.2 ± 1.5	9.4 ± 2.7	20.3 ± 4.7	<0.001
Age (years)	53.9 ± 12	57.1 ± 10.4	64.3 ± 4	0.0356
Male, *n* (%)	59 (58.4%)	35 (71.4%)	4 (66.7%)	0.2965
BMI (kg/m^2^)	25.9 ± 4.6	28.5 ± 5	28.9 ± 4.6	0.0048
Serum creatinine concentration, (mg/dL)	1.39 ± 0.46	1.58 ± 0.64	1.67 ± 0.33	0.044
eGFR (mL/min/1.73 m^2^)	55.3 ± 15.5	50.7 ± 16.3	42.2 ± 10.9	0.0503
Duration between study visit and transplantation (months)	106 ± 64	115 ± 677	95 ± 43	0.65
Duration between study visit and RRT initiation, (months)	157 ± 82	164 ± 73	141 ± 54	0.68
Prior diabetes mellitus, *n* (%)	17 (16.8%)	13 (26.5%)	4 (66.7%)	0.0101
Prior heart disease, *n* (%)	22 (21.8%)	25 (51%)	5 (83.3%)	<0.0001
Charlson comorbidity index	4.1 ± 1.7	4.7 ± 1.7	6.8 ± 1.5	0.0005

BMI—body mass index; eGFR—estimated glomerular filtration rate; RRT—renal replacement therapy.

**Table 3:** Ordinal logistic (univariate and multivariate) regression (*p* < 0.05) of the selected variables and the risk of lung congestion.

	Univariate			Multivariate		
Risk Factor	OR	95% CI	*p*	OR	95% CI	*p*
Charlson comorbidity index	1.21	(1.03,1.44)	0.0233	1.07	(0.89,1.29)	0.4537
Heart disease	2.45	(1.31,4.58)	0.0049	1.24	(0.61,2.50)	0.5423
Symptoms of volume overload	7.50	(3.32,17.0)	<0.0001	5.90	(2.43,14.3)	0.0001
BMI	1.09	(1.03,1.16)	0.0026	1.09	(1.03,1.17)	0.0046
eGFR	0.98	(0.96,0.99)	0.0057	1.29	(0.51,3.26)	0.5721
Uric acid	1.44	(1.16,1.78)	0.0009	0.97	(0.94,1.01)	0.1132
Cholesterol	0.993	(0.988,0.999)	0.0160	0.994	(0.988,1.000)	0.0452
Donor age	1.03	(1.01,1.05)	0.0122	1.01	(0.99,1.03)	0.4657

BMI—body mass index; eGFR—estimated glomerular filtration rate.

**Table 4:** Characteristics of patients in clusters (mean ± st. dev. and %) with *p*-values (<0.05 and <0.1).

Risk Factor	*n* = 61	*n* = 18	*n* = 32	*n* = 45	*p*-Value
Charlson comorbidity index	3.36 ± 0.88	2.83 ± 0.79	4.28 ± 1.20	6.44 ± 1.49	<0.0001
BMI	27.5 ± 5.22	20.9 ± 2.03	29.2 ± 3.54	26.6 ± 4.06	<0.0001
Cholesterol	251 ± 54.3	212 ± 32.8	166 ± 17.0	222 ± 38.5	<0.0001
Males	59%	56%	34%	38%	0.0555
Age	51.0 ± 10.1	45.6 ± 10.2	56.2 ± 10.9	64.4 ± 7.5	<0.0001
Heart disease	18%	17%	47%	51%	0.0004
Diabetes	13%	0%	28%	38%	0.0013
Symptoms of volume overload	8%	11%	13%	29%	0.0261
USBLs	3.9 ± 3.6	2.3 ± 2.3	6.5 ± 4.3	7.0 ± 6.6	0.0003
Presence of AVF	41%	44%	66%	62%	0.0539
AVF burden	80.1 ± 58.9	83.3 ± 86.6	108.1 ± 69.7	120.8 ± 83.3	0.0247
Weight	80.4 ± 16.5	61.7 ± 11.3	87.3 ± 12.7	76.4 ± 12.9	<0.0001
Waist	94.2 ± 13.5	78.4 ± 10.1	102 ± 12.3	96.0 ± 12.5	<0.0001
Serum creatinine	1.45 ± 0.42	1.59 ± 0.76	1.43 ± 0.40	1.45 ± 0.61	0.7558
eGFR	54.0 ± 16.0	50.4 ± 17.7	53.7 ± 14.5	53.4 ± 16.2	0.8715
Platelets	239 ± 66.6	216 ± 37.0	205 ± 51.7	198 ± 53.9	0.0021
Uric acid	7.12 ± 1.14	6.25 ± 1.37	7.21 ± 1.36	6.90 ± 1.42	0.0598
Total protein	7.22 ± 0.45	7.16 ± 0.50	7.00 ± 0.48	6.94 ± 0.53	0.0176
Albumin	4.33 ± 0.26	4.34 ± 0.26	4.28 ± 0.32	4.15 ± 0.30	0.0060
HDL cholesterol	63.0 ± 17.7	61.9 ± 10.6	52.5 ± 10.7	63.8 ± 15.1	0.0061
LDL cholesterol	153 ± 43.4	127 ± 28.4	85.6 ± 22.5	123 ± 33.0	<0.0001

AVF—arteriovenous fistula; BMI—body mass index; eGFR—estimated glomerular filtration rate; US-BLs—ultrasound B-lines.

## Data Availability

The data presented in this study are available on request from the corresponding author.

## References

[1] Lichtenstein D., Mézière G., Biderman P., Gepner A., Barré O. (1997). The comet-tail artifact. An ultrasound sign of alveolar-interstitial syndrome. Am. J. Respir. Crit. Care Med..

[2] Lichtenstein D., Goldstein I., Mourgeon E., Cluzel P., Grenier P., Rouby J.-J. (2004). Comparative diagnostic performances of auscultation, chest radiography, and lung ultrasonography in acute respiratory distress syndrome. Anesthesiology.

[3] Lichtenstein D.A. (2015). BLUE-protocol and FALLS-protocol: Two applications of lung ultrasound in the critically ill. Chest.

[4] Picano E., Frassi F., Agricola E., Gligorova S., Gargani L., Mottola G. (2006). Ultrasound lung comets: A clinically useful sign of extravascular lung water. J. Am. Soc. Echocardiogr..

[5] Gargani L., Pang P.S., Frassi F., Miglioranza M., Dini F.L., Landi P., Picano E. (2015). Persistent pulmonary congestion before discharge predicts rehospitalization in heart failure: A lung ultrasound study. Cardiovasc. Ultrasound.

[6] Öhman J., Harjola V.-P., Karjalainen P., Lassus J. (2018). Focused echocardiography and lung ultrasound protocol for guiding treatment in acute heart failure. ESC Heart Fail..

[7] Zoccali C., Torino C., Tripepi R., Tripepi G., D’Arrigo G., Postorino M., Gargani L., Sicari R., Picano E., Mallamaci F. (2013). Pulmonary congestion predicts cardiac events and mortality in ESRD. J. Am. Soc. Nephrol..

[8] Zoccali C., Torino C., Mallamaci F., Sarafidis P., Papagianni A., Ekart R., Hojs R., Klinger M., Letachowicz K., Fliser D. (2021). A randomized multicenter trial on a lung ultrasound-guided treatment strategy in patients on chronic hemodialysis with high cardiovascular risk. Kidney Int..

[9] Volpicelli G., Lamorte A., Villén T. (2020). What’s new in lung ultrasound during the COVID-19 pandemic. Intensiv. Care Med..

[10] Covic A., Siriopol D., Voroneanu L. (2018). Use of Lung Ultrasound for the Assessment of Volume Status in CKD. Am. J. Kidney Dis..

[11] Marino F., Martorano C., Tripepi R., Bellantoni M., Tripepi G., Mallamaci F., Zoccali C. (2016). Subclinical pulmonary congestion is prevalent in nephrotic syndrome. Kidney Int..

[12] Ciumanghel A., Siriopol I., Blaj M., Siriopol D., Gavrilovici C., Covic A. (2018). B-lines score on lung ultrasound as a direct measure of respiratory dysfunction in ICU patients with acute kidney injury. Int. Urol. Nephrol..

[13] Panuccio V., Tripepi R., Parlongo G., Mafrica A., Caridi G., Catalano F., Marino F., Tripepi G., Mallamaci F., Zoccali C. (2020). Lung ultrasound to detect and monitor pulmonary congestion in patients with acute kidney injury in nephrology wards: A pilot study. J. Nephrol..

[14] Mottola C., Girerd N., Coiro S., Lamiral Z., Rossignol P., Frimat L., Girerd S. (2018). Evaluation of Subclinical Fluid Overload Using Lung Ultrasound and Estimated Plasma Volume in the Postoperative Period Following Kidney Transplantation. Transplant. Proc..

[15] Vanderweckene P., Weekers L., Lancellotti P., Jouret F. (2018). Controversies in the management of the haemodialysis-related arteriovenous fistula following kidney transplantation. Clin. Kidney J..

[16] Malik J., Valerianova A., Tuka V., Trachta P., Bednarova V., Hruskova Z., Slavikova M., Rosner M.H., Tesar V. (2021). The effect of high-flow arteriovenous fistulas on systemic haemodynamics and brain oxygenation. ESC Heart Fail..

[17] Valerianova A., Malik J., Janeckova J., Kovarova L., Tuka V., Trachta P., Lachmanova J., Hladinova Z., Hruskova Z., Tesar V. (2021). Reduction of arteriovenous access blood flow leads to biventricular unloading in haemodialysis patients. Int. J. Cardiol..

[18] Mudoni A., Caccetta F., Caroppo M., Musio F., Accogli A., Zacheo M.D., Burzo M.D., Gallieni M., Nuzzo V. (2016). Echo color Doppler ultrasound: A valuable diagnostic tool in the assessment of arteriovenous fistula in hemodialysis patients. J. Vasc. Access.

[19] Jambrik Z., Monti S., Coppola V., Agricola E., Mottola G., Miniati M., Picano E. (2004). Usefulness of ultrasound lung comets as a nonradiologic sign of extravascular lung water. Am. J. Cardiol..

[20] Gargani L., Volpicelli G. (2014). How I do it: Lung ultrasound. Cardiovasc. Ultrasound.

[21] Gargani L., Sicari R., Raciti M., Serasini L., Passera M., Torino C., Letachowicz K., Ekart R., Fliser D., Covic A. (2016). Efficacy of a remote web-based lung ultrasound training for nephrologists and cardiologists: A LUST trial sub-project. Nephrol. Dial. Transplant..

[22] Abreo K., Sachdeva B., Abreo A.P. (2021). To ligate or not to ligate hemodialysis arteriovenous fistulas in kidney transplant patients. J. Vasc. Access.

[23] Letachowicz K., Banasik M., Królicka A., Mazanowska O., Gołębiowski T., Augustyniak-Bartosik H., Zmonarski S., Kamińska D., Kuriata-Kordek M., Krajewska M. (2021). Vascular Access Perspectives in Patients After Kidney Transplantation. Front. Surg..

[24] Torino C., Gargani L., Sicari R., Letachowicz K., Ekart R., Fliser D., Covic A., Siamopoulos K., Stavroulopoulos A., Massy Z.A. (2020). Inflammation is an amplifier of lung congestion by high lv filling pressure in hemodialysis patients: A longitudinal study. J. Nephrol..

[25] Cluster: Cluster Analysis Basics and Extensions R Package Version 2.1.2. https://cran.rproject.org/web/packages/cluster/cluster.pdf.

[26] R Core Team (2021). A Language and Environment for Statistical Computing, version 4.1.2.

[27] Marczewski E., Steinhaus H. (1958). On a certain distance of sets and the corresponding distance of functions. Colloq. Math..

[28] Tukiendorf A., Kaźmierski R., Michalak S. (2013). The taxonomy statistic uncovers novel clinical patterns in a population of ischemic stroke patients. PLoS ONE.

[29] Miglioranza M.H., Gargani L., Sant’Anna R.T., Rover M.M., Martins V.M., Mantovani A., Weber C., Moraes M.A., Feldman C.J., Kalil R.A.K. (2013). Lung ultrasound for the evaluation of pulmonary congestion in outpatients: A comparison with clinical assessment, natriuretic peptides, and echocardiography. JACC Cardiovasc. Imaging.

[30] Picano E., Scali M.C. (2017). The lung water cascade in heart failure. Echocardiography.

[31] Dwyer K.H., Merz A., Lewis E.F., Claggett B.L., Crousillat D.R., Lau E.S., Silverman M.B., Peck J., Rivero J., Cheng S. (2018). Pulmonary Congestion by Lung Ultrasound in Ambulatory Patients with Heart Failure with Reduced or Preserved Ejection Fraction and Hypertension. J. Card. Fail..

[32] Torino C., Tripepi R., Loutradis C., Sarafidis P., Tripepi G., Mallamaci F., Zoccali C. (2021). Can the assessment of ultrasound lung water in haemodialysis patients be simplified?. Nephrol. Dial. Transplant..

[33] Palazzuoli A., Ruocco G., Franci B., Evangelista I., Lucani B., Nuti R., Pellicori P. (2020). Ultrasound indices of congestion in patients with acute heart failure according to body mass index. Clin. Res. Cardiol..

[34] Brainin P., Claggett B., Lewis E.F., Dwyer K.H., Merz A.A., Silverman M.B., Swamy V., Biering-Sørensen T., Rivero J., Cheng S. (2020). Body mass index and B-lines on lung ultrasonography in chronic and acute heart failure. ESC Heart Fail..

[35] Torino C., Gargani L., Sicari R., Letachowicz K., Ekart R., Fliser D., Covic A., Siamopoulos K., Stavroulopoulos A., Massy Z.A. (2016). The Agreement between Auscultation and Lung Ultrasound in Hemodialysis Patients: The LUST Study. Clin. J. Am. Soc. Nephrol..

[36] Coiro S., Rossignol P., Ambrosio G., Carluccio E., Alunni G., Murrone A., Tritto I., Zannad F., Girerd N. (2015). Prognostic value of residual pulmonary congestion at discharge assessed by lung ultrasound imaging in heart failure. Eur. J. Heart. Fail..

[37] Platz E., Merz A., Jhund P., Vazir A., Campbell R., Mcmurray J. (2017). Dynamic changes and prognostic value of pulmonary congestion by lung ultrasound in acute and chronic heart failure: A systematic review. Eur. J. Heart Fail..

[38] Miglioranza M.H., Picano E., Badano L., Sant’Anna R., Rover M., Zaffaroni F., Sicari R., Kalil R.K., Leiria T.L., Gargani L. (2017). Pulmonary congestion evaluated by lung ultrasound predicts decompensation in heart failure outpatients. Int. J. Cardiol..

[39] Platz E., Campbell R.T., Claggett B., Lewis E.F., Groarke J.D., Docherty K., Lee M., Merz A., Silverman M., Swamy V. (2019). Lung Ultrasound in Acute Heart Failure: Prevalence of Pulmonary Congestion and Short- and Long-Term Outcomes. JACC Heart Fail..

[40] Gargani L., Pugliese N.R., Frassi F., Frumento P., Poggianti E., Mazzola M., De Biase N., Landi P., Masi S., Taddei S. (2021). Prognostic value of lung ultrasound in patients hospitalized for heart disease irrespective of symptoms and ejection fraction. ESC Heart Fail..

[41] Šrajer L.L., Marko K., Hojs N.V., Piko N., Bevc S., Hojs R., Ekart R. (2021). Lung ultrasound, hemoglobin, and NT-proBNP in peritoneal dialysis patients. Clin. Nephrol..

[42] Saad M.M., Kamal J., Moussaly E., Karam B., Mansour W., Gobran E., Abbasi S.H., Mahgoub A., Singh P., Hardy R. (2018). Relevance of B-Lines on Lung Ultrasound in Volume Overload and Pulmonary Congestion: Clinical Correlations and Outcomes in Patients on Hemodialysis. Cardiorenal Med..

[43] Pellicori P., Shah P., Cuthbert J., Urbinati A., Zhang J., Kallvikbacka-Bennett A., Clark A.L., Cleland J.G. (2019). Prevalence, pattern and clinical relevance of ultrasound indices of congestion in outpatients with heart failure. Eur. J. Heart Fail..

[44] Rao N.N., Stokes M.B., Rajwani A., Ullah S., Williams K., King D., Macaulay E., Russell C.H., Olakkengil S., Carroll R.P. (2019). Effects of Arteriovenous Fistula Ligation on Cardiac Structure and Function in Kidney Transplant Recipients. Circulation.

[45] Hetz P., Pirklbauer M., Müller S., Posch L., Gummerer M., Tiefenthaler M. (2020). Prophylactic Ligature of AV Fistula Prevents High Output Heart Failure after Kidney Transplantation. Am. J. Nephrol..

[46] Golper T.A., Hartle P.M., Bian A. (2015). Arteriovenous fistula creation may slow estimated glomerular filtration rate trajectory. Nephrol. Dial. Transplant..

[47] Weekers L., Vanderweckene P., Castanares-Zapatero D., Bonvoisin C., Hamoir E., Maweja S., Krzesinski J.-M., Delanaye P., Pottel H., Jouret F. (2017). The closure of arteriovenous fistula in kidney transplant recipients is associated with an acceleration of kidney function decline. Nephrol. Dial. Transplant..

[48] Bardowska K., Letachowicz K., Kamińska D., Kusztal M., Gołębiowski T., Królicki T., Zajdel K., Mazanowska O., Janczak D., Krajewska M. (2020). The attitude of kidney transplant recipients towards elective arteriovenous fistula ligation. PLoS ONE.

[49] Trampuž B.V., Arnol M., Gubenšek J., Ponikvar R., Ponikvar J.B. (2021). A national cohort study on hemodialysis arteriovenous fistulas after kidney transplantation–Long-term patency, use and complications. BMC Nephrol..

[50] Letachowicz K., Kusztal M., Gołębiowski T., Letachowicz W., Weyde W., Klinger M. (2016). External dilator-assisted banding for high-flow hemodialysis arteriovenous fistula. Ren. Fail..

[51] Bojakowski K., Gziut A., Góra R., Foroncewicz B., Kaźmierczak S., Kasprzak D., Małyszko J., Andziak P. (2021). To Close, Observe, or Reconstruct: The Third Way of Managing Dialysis Fistula Aneurysms in Kidney Transplant Recipients. J. Clin. Med..

[52] Iwakura K., Onishi T. (2021). A practical guide to the lung ultrasound for the assessment of congestive heart failure. J. Echocardiogr..

[53] Yang F., Wang Q., Zhi G., Zhang L., Huang D., Dangsheng H., Zhang M. (2017). The application of lung ultrasound in acute decompensated heart failure in heart failure with preserved and reduced ejection fraction. Echocardiography.

